# Residual-Guided Hybrid Stochastic Modeling: A Two-Stage Learning Framework for Urban GNSS Positioning Enhancement

**DOI:** 10.3390/s26175622

**Published:** 2026-09-04

**Authors:** Juan Yin, Wenqiang Li, Ruichang Fan, Yue Yuan, Zhiheng Zhao, Dingjie Xu, Zhidong Yang, Feng Shen

**Affiliations:** 1 School of Instrumentation Science and Engineering, Harbin Institute of Technology, Harbin 150001, China; 20b901028@stu.hit.edu.cn (J.Y.); liwenq.1996@gmail.com (W.L.); f1446272188@163.com (R.F.); xdj1966@hit.edu.cn (D.X.); 2 Department of Industrial and Systems Engineering, The Hong Kong Polytechnic University, Hong Kong SAR 999077, China; yuena.yuan@polyu.edu.hk (Y.Y.); zhiheng.zhao@polyu.edu.hk (Z.Z.)

**Keywords:** GNSS stochastic model, pseudo-range correction, multipath and non-line-of-sight error, urban positioning, deep learning

## Abstract

The Global Navigation Satellite System (GNSS) has been widely adopted in navigation applications due to its high accuracy and convenience. However, in urban canyon environments, severe signal blockage caused by buildings and trees introduces substantial non-line-of-sight errors and multipath effects, leading to degraded and highly fluctuating positioning performance. To address this issue, this paper proposes a residual-guided hybrid stochastic modeling framework. The method operates in two stages: first, a pseudo-range correction estimation network takes GNSS parameters containing residuals as input to estimate pseudo-range correction; second, these estimated corrections together with elevation angle and carrier-to-noise ratio are fed into a hybrid stochastic model parameter estimation network to determine model parameters. This design adjusts the pseudo-range observations to reduce the positioning loss without requiring true pseudo-range errors, which are difficult to obtain in real-world scenarios. Meanwhile, the explicit modeling of relationships among pseudo-range correction, elevation angle, and carrier-to-noise ratio renders the stochastic model parameters interpretable. Experiments on public urban GNSS datasets demonstrate that the proposed method achieves competitive positioning performance against both conventional and learning-based baselines. It delivers notable accuracy improvements in light urban canyon environments, particularly on the KLT2 sequence, while maintaining robust and competitive performance in the more challenging TST and Mong Kok scenarios. These results validate the effectiveness of jointly estimating pseudo-range corrections and adaptive observation weights for enhancing positioning accuracy and robustness across diverse urban environments.

## 1. Introduction

The Global Navigation Satellite System (GNSS) provides high-precision positioning services in open-sky environments and has been widely adopted as the primary positioning source for various navigation applications [1,2]. However, in urban canyons, GNSS signals are subjected to diffraction, reflection, and even severe obstruction by high-rise buildings and trees, thereby inducing significant non-line-of-sight (NLOS) errors and multipath effects, which ultimately degrade observation quality and considerably increase positioning errors [3,4,5]. A variety of studies have been conducted to mitigate the above issues and enhance GNSS positioning performance in urban scenarios.

Traditional approaches to improving GNSS positioning performance primarily optimize the Weighted Least Squares solution structure through measurement enhancement and downweighting low-quality signals [6,7,8]. For measurement enhancement, refined mathematical models are typically designed by introducing raw GNSS observations combined with atmospheric delay correction models to mitigate the impact of systematic errors on positioning results. For instance, the Klobuchar ionospheric model is suitable for ionospheric delay correction for single-frequency users [9]. In addition to atmospheric corrections, high-precision GNSS processing also relies on satellite clock products and antenna phase-center models. Previous studies have shown that satellite clock interpolation and antenna phase-center corrections can affect observation residuals and coordinate solutions [10,11]. These products and models are used to compensate for error sources that can be described by established models or external correction information. Regarding weight determination, the carrier-to-noise ratio (C/N_0_) and elevation angle are commonly adopted as core reference indicators, with different types of stochastic models employed to assess satellite observation quality [12]. Euler et al. proposed an elevation-dependent exponential weighting model that reflects signal quality differences through satellite geometry variations [13]. Hartinger et al. established the SIGMA-ε model that directly uses C/N_0_ to determine observation variance [14], and Zhang et al. developed a composite stochastic model combining elevation angle, azimuth, and C/N_0_, which demonstrates superior adaptability in complex terrain environments such as canyons [15]. However, approaches based on physical models and empirical formulas exhibit satisfactory interpretability but fail to maintain stable and effective performance in complex scenarios.

Furthermore, to alleviate the adverse impact of NLOS on GNSS positioning, prior research has leveraged auxiliary information from inertial navigation systems (INSs) [16], visual cameras [17], LiDAR [18], and 3D city maps [19] to enhance positioning performance. INSs provide high-frequency and stable short-term positioning, and maintain continuous navigation when GNSS signals are unavailable, thus being commonly applied in integrated navigation systems [20]. Low-cost MEMS inertial sensors suffer from rapid error drift, while high-grade INSs will greatly increase system cost and size. Fisheye cameras detect surrounding obstacles and extract sky visibility data to distinguish LOS and NLOS signals, which helps improve GNSS observation reliability in urban environments [21]. However, visual sensors are easily affected by light, weather and dynamic occlusions, restricting their robustness in complex outdoor conditions. LiDAR can mitigate GNSS NLOS errors, but with limited effectiveness [22]. Three-dimensional city models assist positioning through shadow matching and ray tracing to quantify NLOS propagation delays, which can effectively suppress NLOS errors without additional sensors [23,24]. Nevertheless, high-precision 3D map construction and real-time updating are difficult, and positioning performance degrades sharply in unmarked or dynamically changing areas. Although external sensor-aided methods improve positioning robustness, they are restricted by high costs and environmental sensitivity, making it difficult to meet the requirements of low-cost and large-scale applications.

Benefiting from the powerful nonlinear fitting and implicit feature mining capabilities, learning-based data-driven methods have gradually become a mainstream scheme to break through the performance bottleneck of traditional GNSS positioning in complex urban environments [25,26,27,28]. Existing learning-based approaches are mainly divided into two categories: satellite observation quality classification and measurement error prediction. The former applies machine learning and deep learning to distinguish different signal categories by fusing GNSS features and multi-sensor data [29,30]. The latter constructs model networks to predict pseudo-range error, so as to improve the overall observation quality [31,32]. Unlike traditional methods, learning-based methods require no additional hardware, adapt to complex error distributions, and possess superior environmental adaptability. In related integrated navigation research, deep learning has also been introduced to adaptively model measurement uncertainty and improve navigation performance under GNSS degradation [33], further demonstrating the potential of data-driven methods for adaptive stochastic modeling.

However, current learning-based methods still face critical limitations in that most models lack physical constraints, leading to weak interpretability, while model training relies on a large amount of high-quality labeled data, which is costly to acquire in real urban scenarios.

To address the aforementioned problems, this paper proposes a residual-guided hybrid stochastic modeling framework, a two-stage learning architecture for urban GNSS positioning enhancement. The proposed method organically integrates pseudo-range correction and adaptive stochastic modeling, retaining the interpretability of physical stochastic models while leveraging the nonlinear fitting capability of deep learning. In the first stage, a residual-guided pseudo-range correction estimation network is designed to achieve pseudo-range correction by fusing multi-dimensional GNSS features, realizing implicit calibration of observations. In the second stage, an adaptive parameter estimation network is developed to learn optimal coefficients for the hybrid stochastic model combining elevation angle and C/N_0_, enabling dynamic parameter tuning and overcoming the inflexibility of fixed empirical coefficients. By combining correction and adaptive weighting, the proposed method effectively improves both positioning accuracy and robustness in urban canyons.

The main contributions of this work are summarized as follows:

1. A residual-guided pseudo-range correction network is designed to estimate corrections, which are applied to pseudo-range observations to improve positioning accuracy and used as error-related features for adaptive stochastic modeling.
2. An adaptive parameter estimation network is developed to learn optimal coefficients for the hybrid stochastic model combining elevation angle and C/N_0_, allowing dynamic parameter tuning and overcoming the limitations of fixed empirical coefficients.
3. The proposed method organically integrates pseudo-range correction and adaptive stochastic modeling, preserving the interpretability of physical models while utilizing the nonlinear fitting capability of deep learning. Comprehensive experiments on public urban datasets validate the effectiveness and robustness of the proposed method.

## 2. Methods

**Figure 1:**
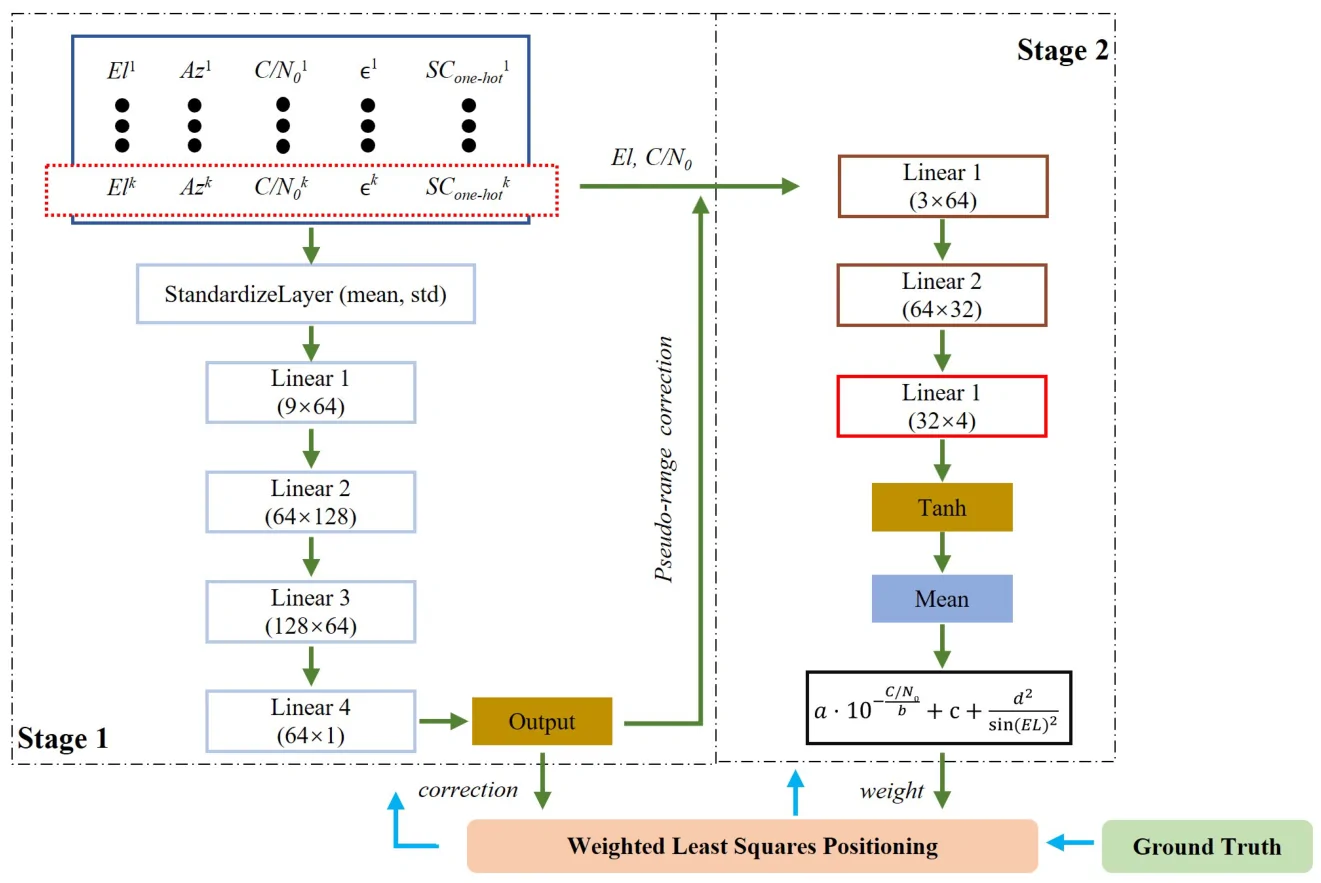
Framework of the proposed residual-guided hybrid stochastic modeling method.

The proposed method adopts a two-stage architecture as shown in Figure 1. Stage 1 is a residual-guided pseudo-range correction estimation network. It takes satellite elevation angle, azimuth, C/N_0_, residuals, and satellite constellation one-hot codes as inputs, standardizes the features, and passes them through fully connected layers to output the estimated pseudo-range correction. Stage 2 is a hybrid stochastic model parameter estimation network. It receives elevation angle, C/N_0_, and the pseudo-range correction estimated from Stage 1, then outputs the parameters of the hybrid stochastic model through connected layers and a mean pooling operation. These parameters determine the observation weights for the subsequent weighted least squares positioning module.

During training, the two stages operate sequentially. Stage 1 first estimates the pseudo-range correction, which together with elevation angle and C/N_0_ feeds into Stage 2 to produce the hybrid stochastic model parameters.

The weighted least squares module then computes the position solution using these parameters and pseudo-range correction, and the positioning error is back-propagated to update both networks. During inference, raw GNSS observations are processed through the trained networks to obtain pseudo-range correction and observation weights, followed by weighted least squares positioning.

This architecture enhances GNSS positioning through two mechanisms: the pseudo-range correction network adjusts observations, and the hybrid stochastic model adaptively assigns reliable weights to individual satellites. The combination of pseudo-range correction estimation and adaptive weighting improves both accuracy and robustness in urban canyon environments.

### 2.1. GNSS Positioning

The standard GNSS pseudo-range observation follows

(1) p=r+c∆t+I+T+ε

Here, *p* is the pseudo-range, *c* is the speed of light, *I* and *T* are the ionospheric and tropospheric delays, ∆t is the receiver’s time bias, ε is Gaussian noise, and *r* is the distance between the satellite and the receiver.

When there are *n* observations, the observation equations are

(2) $$\tilde{Z}=h\left(y\right)=\left[\begin{array}{c} r_{1}+c\Delta t+I_{1}+T_{1}\\ r_{2}+c\Delta t+I_{2}+T_{2}\\ \ldots \\ r_{n}+c\Delta t+I_{n}+T_{n} \end{array}\right]$$

where $\tilde{Z}={\left[p_{1},p_{2},...,p_{n}\right]}^{T}$ denotes the vector of pseudo-range observations and $y=(x_{r},y_{r},z_{r},\;\Delta t)$ represents the receiver state vector to be estimated, which consists of the 3D position coordinates $\left(x_{r},y_{r},z_{r}\right)$ in the Earth-Centered Earth-Fixed (ECEF) frame and the receiver clock bias ∆t, The unknown *y* state can be optimally solved by the weighted least squares (WLS) method, formulated as

(3) $$y=f_{WLS}\left(W,\tilde{Z}\right)$$

The above compact form can be explicitly expressed as the iterative Gauss–Newton weighted least squares update rule:

(4) $$\Delta y={\left(H^{T}WH\right)}^{-1}H^{T}W\left(\tilde{Z}-h\left(y_{i}\right)\right)$$

(5) $$y_{i+1}=y_{i}+\Delta y$$

where **H** is the Jacobian matrix of the observation function with respect to the receiver state vector **y**, formulated as

(6) $$H=\left[\begin{array}{cccc} \frac{x_{r}-x_{1}}{r_{1}} & \frac{y_{r}-y_{1}}{r_{1}} & \frac{z_{r}-z_{1}}{r_{1}} & c\\ \frac{x_{r}-x_{2}}{r_{2}} & \frac{y_{r}-y_{2}}{r_{2}} & \frac{z_{r}-z_{2}}{r_{2}} & c\\ \vdots & \vdots & \vdots & \vdots \\ \frac{x_{r}-x_{n}}{r_{n}} & \frac{y_{r}-y_{n}}{r_{n}} & \frac{z_{r}-z_{n}}{r_{n}} & c \end{array}\right]$$

However, in urban canyons, NLOS and multipath effects introduce an additional bias *b*. The observation model becomes

(7) p=r+c∆t+I+T+ε+b

The estimated bias corrects pseudo-ranges:

(8) $$\hat{\tilde{Z}}=\tilde{Z}-\hat{b}$$

The corrected measurements and observation weights **W** are then fed into the weighted least squares method:

(9) $$y=f_{WLS}\left(W,\hat{\tilde{Z}}\right)$$

According to the above equation, achieving optimal positioning results relies on the weight **W** and the corrected observed pseudo-range $\hat{\tilde{Z}}$. The bias $\hat{b}$ in the pseudo-range measurements is predicted by the residual-guided pseudo-range correction estimation network described in this section. Meanwhile, the weight matrix **W** is generated by the hybrid stochastic model parameter estimation network.

### 2.2. Pseudo-Range Correction Estimation Network

**Figure 2:**
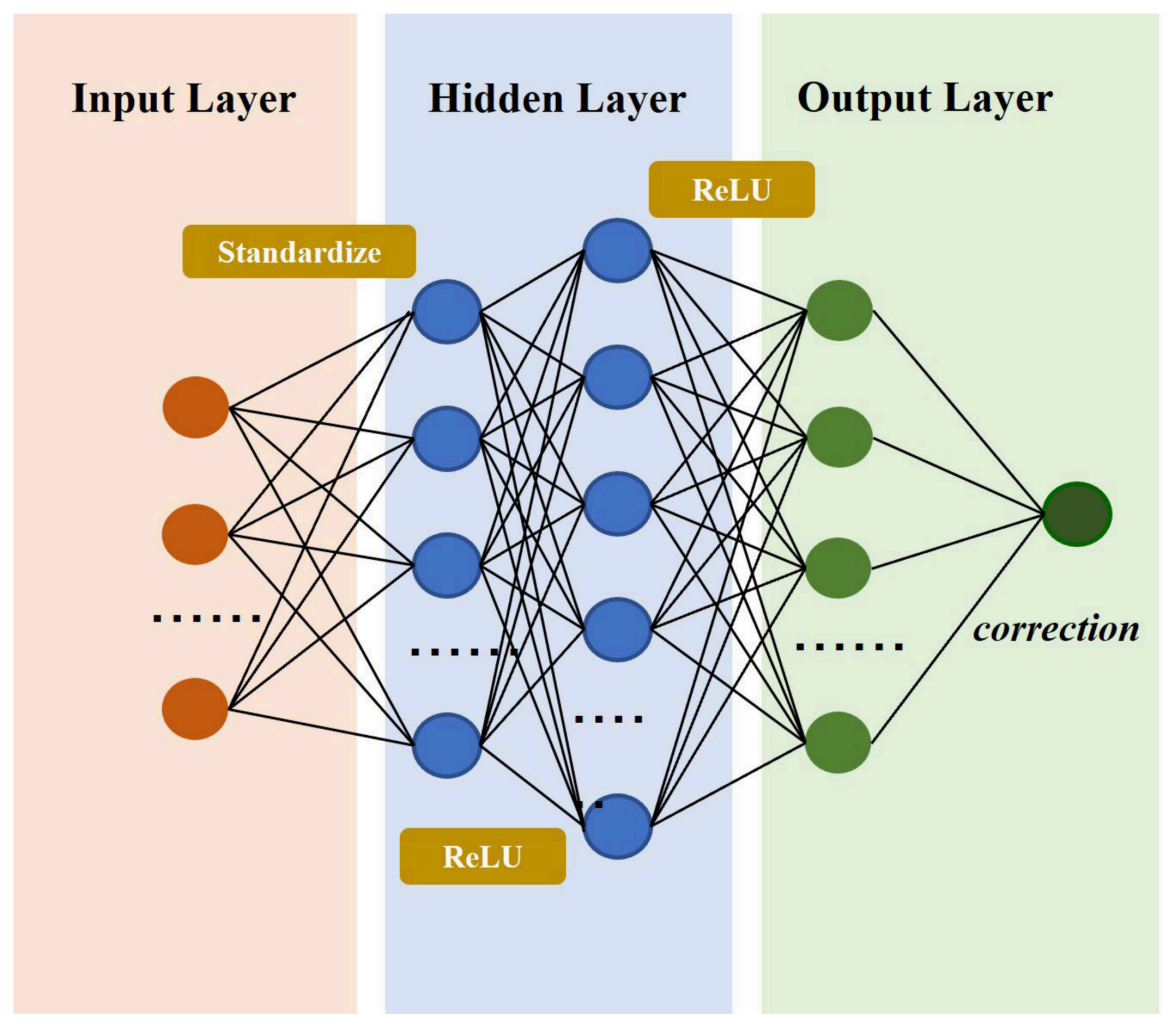
Pseudo-range correction estimation network diagram.

Figure 2 shows the schematic of the proposed pseudo-range correction estimation network. The input features are first standardized, then fed into three fully connected hidden layers. The output layer uses a linear layer to estimate pseudo-range corrections. The network is formulated as

(10) $$\hat{b}=f_{nn,b}\left(X;\Theta \right)$$

where *X* denotes the batch input and Θ the network parameters. The input *X* contains five key features, namely:

- Carrier-to-noise ratio (C/N_0_): A critical metric of signal quality, defined as the ratio of carrier power to noise power per unit bandwidth, typically in dB-Hz.
- Elevation angle: The vertical angle between the receiver’s horizon and the satellite line-of-sight (LOS). Higher-elevation satellites are less prone to obstruction in urban environments.
- Azimuth: The horizontal angle of the satellite relative to the receiver, providing additional geometric context for satellite distribution.
- Equal-weight least squares residuals: Pseudo-range residuals from an initial equal-weight least squares position solution, used to identify potentially unreliable measurements.
- Satellite constellation one-hot codes: These indicate the satellite’s constellation (e.g., GPS, BDS, GLONASS, …) using one-hot encoding, enabling the network to distinguish signals from different systems.

We optimize the measurements with the ground truth position and L2 norm loss, as follows:

(11) $$L=\|y_{gt}-\hat{y}{\|}_{2}$$

(12) $$\frac{\partial L}{\partial \Theta_{b}}=\frac{\partial L}{\partial \hat{y}}\left(\frac{\partial \hat{y}}{\partial \hat{\tilde{z}}}\frac{\partial \hat{\tilde{z}}}{\partial \hat{b}}+\frac{\partial \hat{y}}{\partial W}\frac{\partial W}{\partial \hat{b}}\right)\frac{\partial \hat{b}}{\partial \Theta_{b}}$$

(13) $$\Theta_{n+1}=\Theta_{n}-\eta \left(\frac{\partial L}{\partial \Theta }+\lambda \Theta_{n}\right)$$

To determine the optimal network parameters, we minimize the loss function via gradient backpropagation and AdamW optimization, as detailed in the above equations. Here, η denotes the learning rate and λ represents the weight decay coefficient.

This method takes typical GNSS features such as observation residuals as inputs to guide error identification and quantification, thereby providing a pseudo-range correction for subsequent positioning.

### 2.3. Hybrid Stochastic Model

The stochastic model characterizes the statistical properties of GNSS observation errors and serves as a core component of the positioning mathematical framework. It directly determines observation weights in weighted least squares positioning, thereby governing final positioning accuracy.

Currently, two classical fixed-parameter models are commonly used: the elevation angle model and the C/N_0_ model.

The elevation angle model is built on the principle that GNSS observation errors, primarily from tropospheric delay and low-elevation multipath effects, decrease as the elevation angle increases. Its classical form is defined as

(14) $$\sigma_{EL}^{2}=a_{EL}^{2}+b_{EL}^{2}/\sin^{2}\left(EL\right)$$

where EL is the elevation angle and $a_{EL}$ and $b_{EL}$ are empirical coefficients determined in advance.

This model performs effectively in open environments with mild multipath effects, and adequately suppresses tropospheric residual errors. However, in complex urban canyons, it remains insensitive to signal diffraction and NLOS errors. Such errors frequently occur on high-elevation satellites whose propagation paths are obstructed, leading to unreasonable weight allocation.

The C/N_0_ model adopts C/N_0_ as the core variable, since C/N_0_ directly reflects signal attenuation and distortion during propagation. Its classical form is expressed as

(15) $$\sigma_{C/N_{0}}^{2}=C_{i}\cdot 10^{-\frac{C/N_{0}}{10}}$$

where $C_{i}$ is a constant related to the signal wavelength.

This model correlates more strongly with multipath and NLOS errors than the elevation angle model, and better identifies abnormal observations caused by urban occlusion. However, without geometric constraints, the model cannot effectively distinguish low-elevation satellites with larger atmospheric delays. In complex environments, this deficiency may result in unreasonable weight allocation.

To address the inherent limitations of single-fixed-parameter models, this paper integrates the complementary strengths of elevation angle and C/N_0_ approaches, proposing a hybrid stochastic model formulated as

(16) $$\sigma^{2}=a\cdot 10^{-\frac{C/N_{0}}{b}}+c+\frac{d^{2}}{\sin^{2}\left(EL\right)}$$

where a, b, c, d are adaptive parameters and $\sigma^{2}$ is the pseudo-range observation variance. Unlike classical models with fixed empirical coefficients, this hybrid formulation enables parameter adjustment according to real-time environmental conditions, thereby describing error distributions more accurately across heterogeneous urban scenes and achieving more reasonable observation weights.

### 2.4. Adaptive Parameter Estimation Network

**Figure 3:**
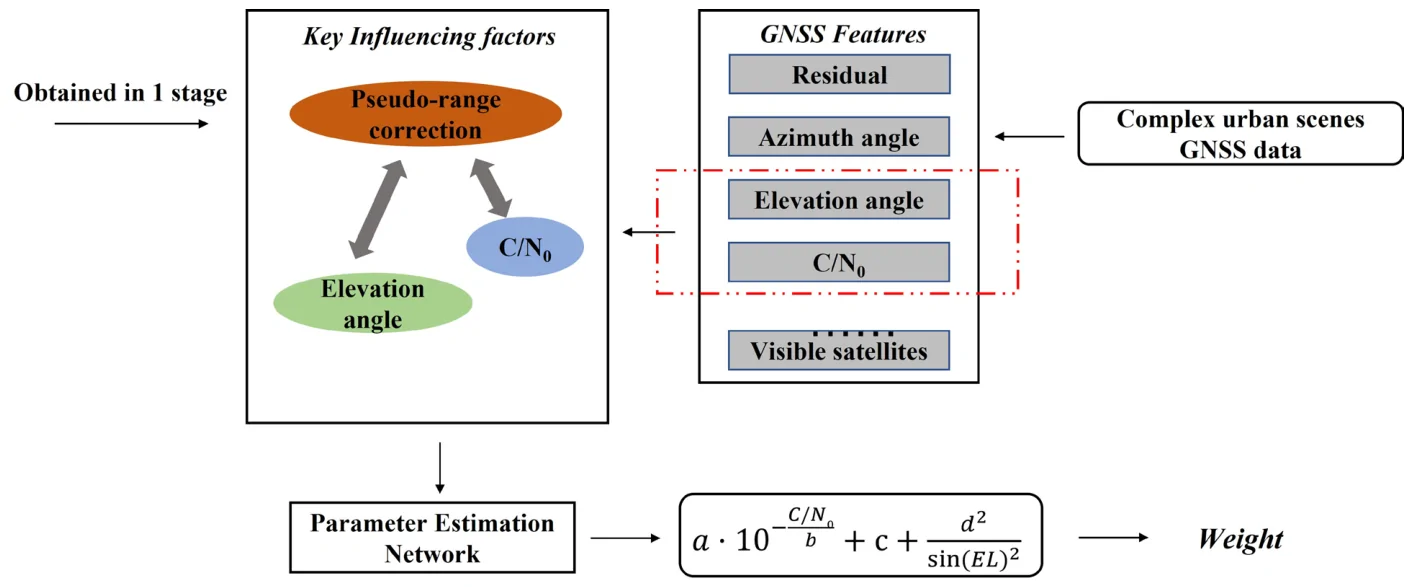
Framework of adaptive parameter estimation for hybrid stochastic model.

For adaptive parameter estimation of the hybrid stochastic model, we design a deep learning-based approach, as illustrated in Figure 3. The network captures the inherent mapping between GNSS observation features and pseudo-range correction characteristics, enabling dynamic optimization of the model parameters.

The parameter estimation network takes C/N_0_, elevation angle, and the pseudo-range correction estimated in Stage 1 as inputs. These features characterize signal quality, geometric propagation path, and observation quality, respectively, providing complementary information for adaptive parameter estimation. Through nonlinear fitting and mean pooling, the network outputs the optimal parameters a, b, c, d of the hybrid stochastic model.

The mean pooling operation is performed over the visible satellites within each epoch. Therefore, changes in the number of visible satellites do not affect the network dimensions. Although the estimated parameters are shared within an epoch, the resulting weights remain unique because they are calculated using each satellite’s own C/N_0_ and elevation angle.

The observation weight is then determined by the inverse variance:

(17) $$w_{i}=\frac{1}{\sigma_{i}^{2}}$$

where $w_{i}$ is the weight of the *i*-th pseudo-range observation. As shown in Equation (18), the gradient chain for the hybrid stochastic model parameter network is

(18) $$\frac{\partial L}{\partial \Theta_{w}}=\frac{\partial L}{\partial \hat{y}}\frac{\partial \hat{y}}{\partial W}\frac{\partial W}{\partial \sigma^{2}}\frac{\partial \sigma^{2}}{\partial \Theta_{w}}$$

where $\Theta_{w}$ denotes the parameters of the hybrid model estimation network. Through this process, adaptive adjustment of the stochastic model is achieved.

The network dynamically updates model parameters according to real-time scene features, subsequently adjusting the weights of individual observations to downweight abnormal measurements affected by multipath and NLOS effects, thereby enhancing the robustness and accuracy of GNSS positioning in complex urban environments.

## 3. Experiment Setup

We tested the model performance in urban canyon environments using two open-source datasets: the KLT Dataset [34] and UrbanNav Dataset [35]. Both contain extensive urban canyon scenarios; five representative trajectories were selected for training and evaluation, with their characteristics summarized in Table 1.

We used KLT3 as the training sequence and KLT1 and KLT2 as the test sequences to evaluate positioning accuracy and trajectory stability in light urban environments. All three sequences were collected on the same day in the Kowloon Tong area using the same platform equipped with a u-blox F9P receiver (u-blox AG, Thalwil, Switzerland). They represent different but spatially adjacent trajectories within a similar urban environment. Therefore, their satellite geometries may share certain similarities but are not identical because signal obstruction varies along the trajectories. Accordingly, KLT1 and KLT2 mainly evaluate within-dataset generalization to unseen trajectories. The TST and Mong Kok sequences from the UrbanNav dataset were further used to evaluate cross-dataset generalization in medium and harsh urban environments, respectively.

The training settings are shown in Table 2. The hyperparameters were empirically selected based on preliminary tuning conducted on the training set. The model was trained for 100 epochs using the AdamW optimizer with a learning rate of 5 × 10^−3^ and weight decay of 1 × 10^−5^. These hyperparameters were fixed for all test sequences. All experiments ran on a computer equipped with an AMD Ryzen 9 8940HX CPU (Advanced Micro Devices, Inc., Santa Clara, CA, USA) and an NVIDIA GeForce RTX 5060 GPU (NVIDIA Corporation, Santa Clara, CA, USA). The loss function was defined as the L2 norm of the 3D positioning error.

We adopt position error as the evaluation metric to quantify localization accuracy. The position error at epoch *k* is defined as

(19) $$e_{k}={\hat{p}}_{k}-p_{k}^{ref}$$

where ${\hat{p}}_{k}$ denotes the estimated position and $p_{k}^{ref}$ denotes the reference ground truth.

The 2D error and 3D error are computed separately as

(20) $$e_{2D}=\sqrt{e_{E}^{2}+e_{N}^{2}}$$

(21) $$e_{3D}=\sqrt{e_{E}^{2}+e_{N}^{2}+e_{U}^{2}}$$

where $e_{E}$, $e_{N}$, and $e_{U}$ represent the east, north, and up components of $e_{k}$, respectively. We report the mean and maximum errors for all test sequences.

## 4. Comparative Experiments

We validate the proposed method through comparison with both deep learning-based and conventional approaches. TDL-GNSS [36] serves as the learning-based baseline. For the conventional methods, we select three representative approaches for comparison: SPP-E (single-point positioning with equal weights, $w_{i}=1$), RTKLIB (version 2.4.3, via pyrtklib 0.2.7) and goGPS [37] (implemented in TASGNSS 0.1.5).

The observation weight model of RTKLIB is given by

(22) $$\sigma^{2}\left(\theta \right)=a^{2}+\frac{b^{2}}{\sin^{2}\theta }$$

(23) $$w=\frac{1}{\sigma^{2}\left(\theta \right)}$$

where a and b are hyperparameters, typically set to 0.3.

The weighting formula of the goGPS method is given by

(24) $$k_{1}\left(s\right)=-\frac{s-s_{1}}{a},k_{2}\left(s\right)=\frac{s-s_{1}}{s_{0}-s_{1}}$$

(25) $$w=\left\{\begin{array}{c} \frac{1}{\sin^{2}\theta }\left(10^{k_{1}\left(s\right)}\left(\left(\frac{A}{10^{k_{1}\left(s_{0}\right)}}-1\right)k_{2}\left(s\right)+1\right)\right),s<s_{1}\\ 1,s\geq s_{1} \end{array}\right.$$

where *s* denotes C/N_0_, θ is the elevation angle, and the hyperparameters are typically set to A = 30, a = 20, s_0_ = 10, and s_1_ = 50.

**Figure 4:**
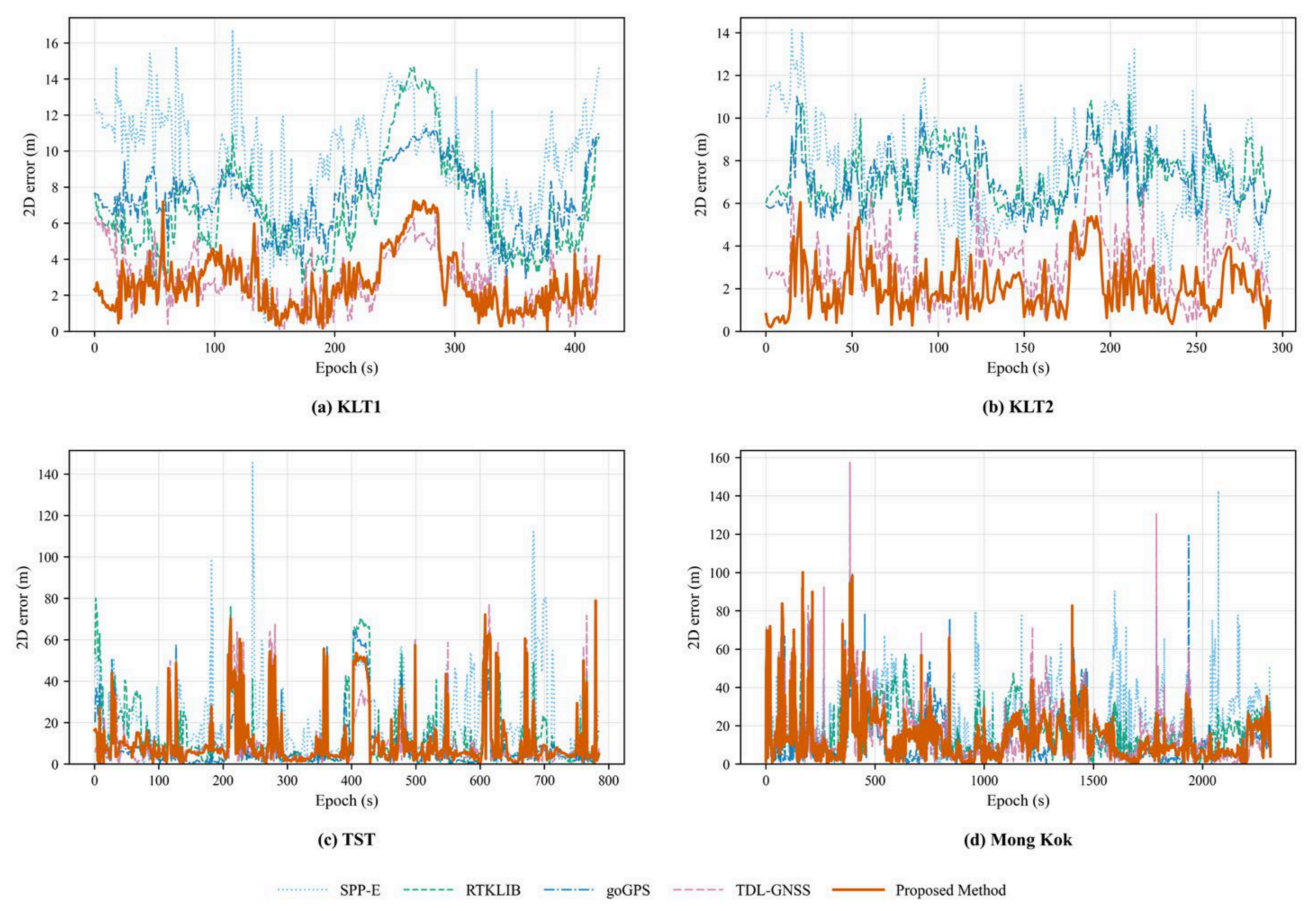
(**a**–**d**) 2D positioning error time series across test sequences.

**Figure 5:**
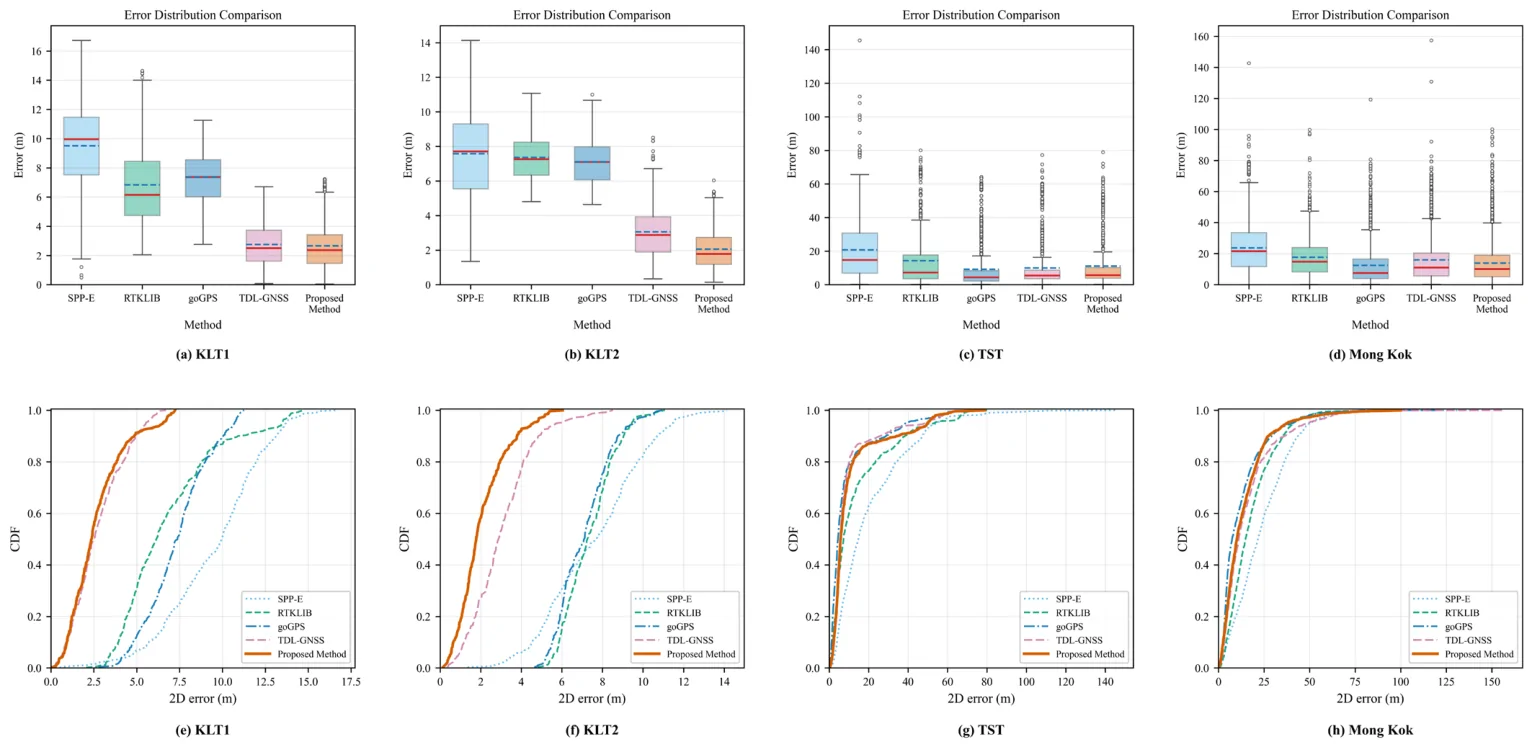
(**a**–**h**) 2D positioning error distribution comparison. In the box plots, the red solid lines indicate the median errors, while the blue dashed lines indicate the mean errors.

We assess the proposed method across four representative test sequences covering varying urban canyon severity. Figure 4 presents the temporal evolution of 2D positioning errors for all compared approaches across the four test sequences. Specifically, Figure 4a,b illustrate the results from KLT1 and KLT2, which are regarded as light urban canyon scenarios, whereas Figure 4c,d show the results from TST and Mong Kok, representing medium and harsh urban canyon conditions, respectively. The statistical results of 2D positioning errors for all five methods are summarized in Table 3. Table 4 and Figure 5 further present the statistical characteristics and distributions of the positioning errors, revealing distinct performance patterns under varying urban canyon conditions.

In the light urban canyon scenarios of KLT1 and KLT2, the proposed method demonstrates competitive accuracy and stable error distributions compared with the four baseline approaches. On KLT1, the proposed method achieves the lowest mean 2D error of 2.66 m and the smallest median error of 2.37 m. Its performance on the remaining error metrics is broadly comparable to that of TDL-GNSS, while it substantially outperforms the three conventional baselines. On KLT2, the proposed method achieves the best performance across all four 2D indicators, with a mean error of 2.06 m, a maximum error of 6.04 m, a median error of 1.78 m, and a 95th-percentile error of 4.59 m. These results indicate that the proposed method can effectively improve 2D positioning accuracy and maintain a compact error distribution in light urban canyon environments.

For the TST sequence, which represents a medium urban canyon scenario, goGPS achieves the best overall 2D positioning performance. As shown by the box plot in Figure 5, the proposed method has a lower median error and a more compact main error distribution than SPP-E and RTKLIB, while its distribution is generally comparable to that of TDL-GNSS. The CDF curves further show that the proposed method reaches high cumulative probabilities earlier than SPP-E and RTKLIB and remains close to TDL-GNSS over most of the error range. Although the proposed method does not achieve the best performance in this sequence, it maintains competitive positioning accuracy after being transferred from the light urban KLT3 training sequence to an independent medium urban dataset.

In the Mong Kok sequence, which represents a harsh urban canyon scenario, all methods exhibit increased errors and intermittent large deviations. The proposed method achieves a mean 2D error of 13.93 m and a median error of 10.10 m, ranking second to goGPS. It also obtains the lowest 95th-percentile error of 37.59 m among all methods, indicating a relatively compact main error distribution. Its maximum error of 100.12 m is comparable to that of RTKLIB and lower than those of SPP-E, goGPS, and TDL-GNSS. These results suggest that the proposed method maintains competitive 2D positioning performance and provides effective high-percentile error control under harsh urban conditions, although isolated large errors remain.

**Figure 6:**
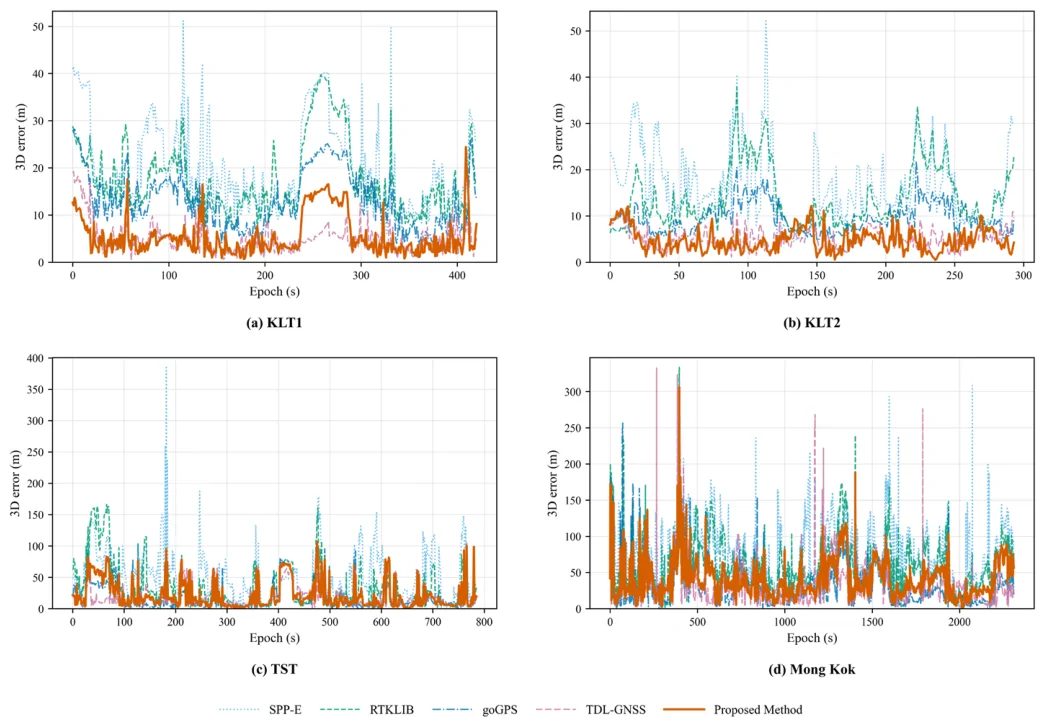
(**a**–**d**) 3D positioning error time series across test sequences.

**Figure 7:**
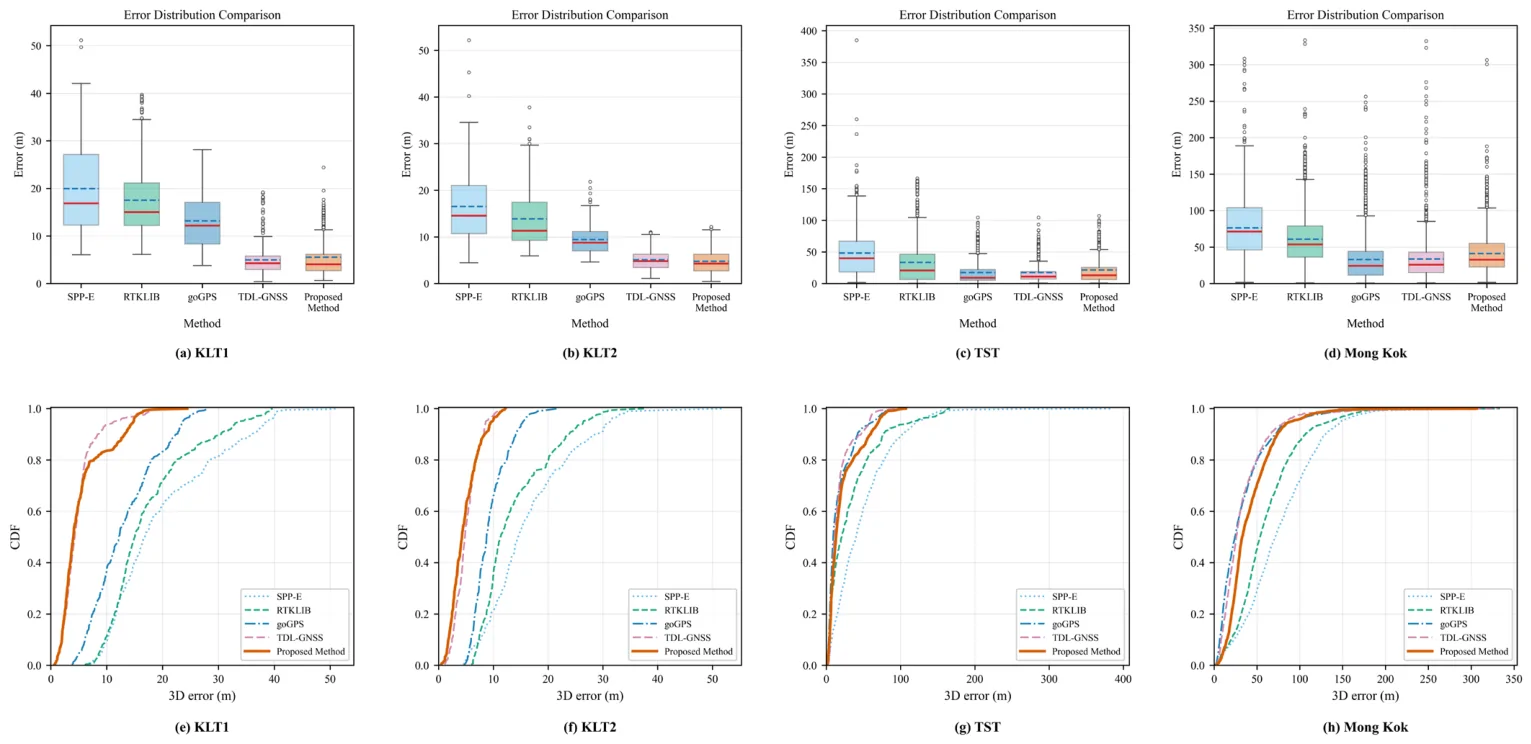
(**a**–**h**) 3D positioning error distribution comparison. In the box plots, the red solid lines indicate the median errors, while the blue dashed lines indicate the mean errors.

Furthermore, Figure 6 and Figure 7, together with Table 5 and Table 6, present the 3D positioning error results. Across the four test sequences, the proposed method maintains competitive 3D positioning performance, with particularly clear advantages in the central error metrics of the light urban canyon sequences.

In the light urban canyon scenarios of KLT1 and KLT2, the proposed method demonstrates favorable 3D positioning accuracy and compact error distributions. On KLT1, it achieves the lowest median error of 4.03 m, indicating accurate positioning for the majority of epochs. For the remaining metrics, the proposed method substantially outperforms the three conventional baselines and remains competitive with TDL-GNSS. On KLT2, it achieves the lowest mean and median errors of 4.74 m and 4.28 m, respectively, while maintaining competitive maximum and 95th-percentile errors. The CDF curve and box plot in Figure 7 further demonstrate its compact central error distribution. These results confirm that the proposed method provides accurate and stable 3D positioning in light urban canyon environments.

For the more challenging TST and Mong Kok sequences, all methods exhibit increased 3D errors because of stronger signal obstruction and multipath effects. Although goGPS and TDL-GNSS generally perform better, the proposed method achieves a similar maximum error on TST and a comparable 95th-percentile error on Mong Kok, while obtaining a lower maximum error than TDL-GNSS on Mong Kok. These results demonstrate that the proposed method maintains competitive 3D positioning performance in complex urban environments.

Overall, the proposed method demonstrates strong 3D positioning performance in light urban canyon environments but shows performance degradation in the more challenging TST and Mong Kok sequences. Since the model is trained only on the light urban KLT3 sequence, the learned relationship between GNSS features, pseudo-range corrections, and observation weights may not fully represent the more complex error characteristics encountered in medium and harsh urban environments. Therefore, although the results demonstrate a certain degree of cross-dataset adaptability, the model’s generalization capability under severe NLOS and multipath conditions requires further improvement.

## 5. Ablation Experiments

**Figure 8:**
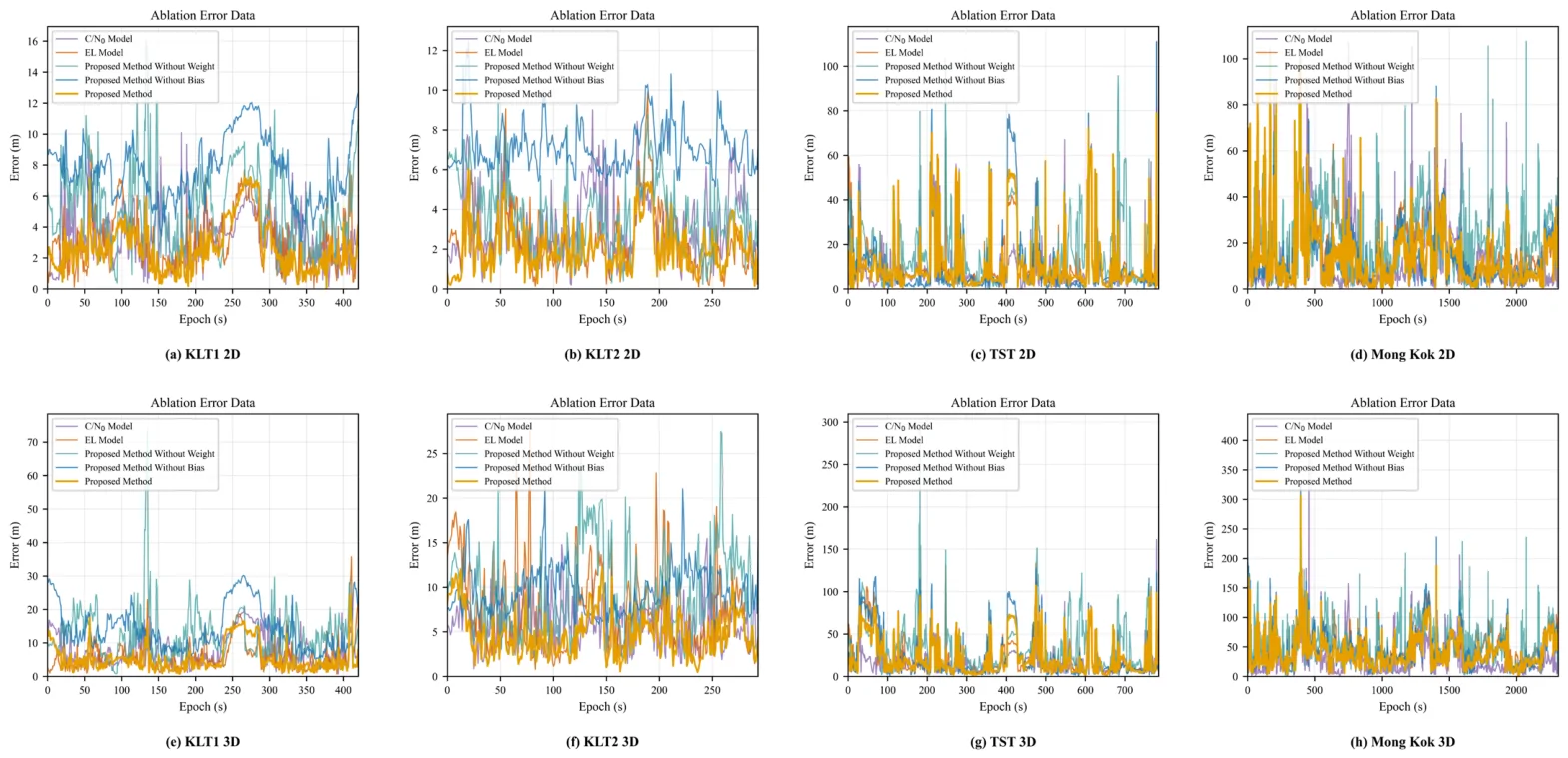
(**a**–**h**) Ablation results of 2D and 3D positioning error.

We conducted ablation experiments on the pseudo-range correction estimation network and the hybrid stochastic modeling strategy to verify the contribution of each component in the proposed framework. The quantitative 2D and 3D positioning results are summarized in Table 7, and the corresponding error variations are shown in Figure 8. Specifically, the C/N_0_ Model replaces the hybrid stochastic model with C/N_0_-based weighting only, the EL Model uses elevation-angle-based weighting only, the Without Bias variant removes pseudo-range correction, and the Without Weight variant replaces adaptive stochastic modeling with equal-weight WLS.

The ablation results for the light urban canyon scenarios of KLT1 and KLT2 show that the complete proposed method generally outperforms the ablation variants. Removing the pseudo-range correction leads to evident performance degradation, demonstrating the contribution of the correction estimation network.

The complete method also generally performs better than the C/N_0_-based and elevation-based models, indicating that combining signal-quality and geometric information provides more effective observation weighting than using either factor alone. The results for the more complex TST and Mong Kok scenarios show that the complete method continues to outperform the Without Bias variant, while the Without Weight variant generally produces larger positioning errors. These findings confirm the contributions of pseudo-range correction and adaptive observation weighting. However, the single-factor models perform better in some individual metrics. A possible reason for this is that the model was trained only on the light urban canyon KLT3 sequence and did not fully learn the more complex obstruction patterns in TST and Mong Kok. In harsh urban environments, elevation angle cannot reliably represent observation quality. Consequently, the learned elevation-related weighting becomes less effective. This indicates that the cross-environment generalization capability of the model still needs improvement.

Overall, the ablation study confirms the contributions of both pseudo-range correction and adaptive stochastic modeling to the proposed framework. The complete method performs particularly well in light urban canyon scenarios and remains effective in more complex environments. However, the varying performance of the weighting strategies indicates that the relationships among elevation angle, signal quality, and observation reliability learned from KLT3 do not fully represent severe urban obstruction conditions. Future work will therefore focus on improving cross-environment generalization by introducing more diverse training data and incorporating additional environmental information to support more reliable pseudo-range correction and observation weighting under severe NLOS and multipath conditions.

## 6. Trajectory Visualization

**Figure 9:**
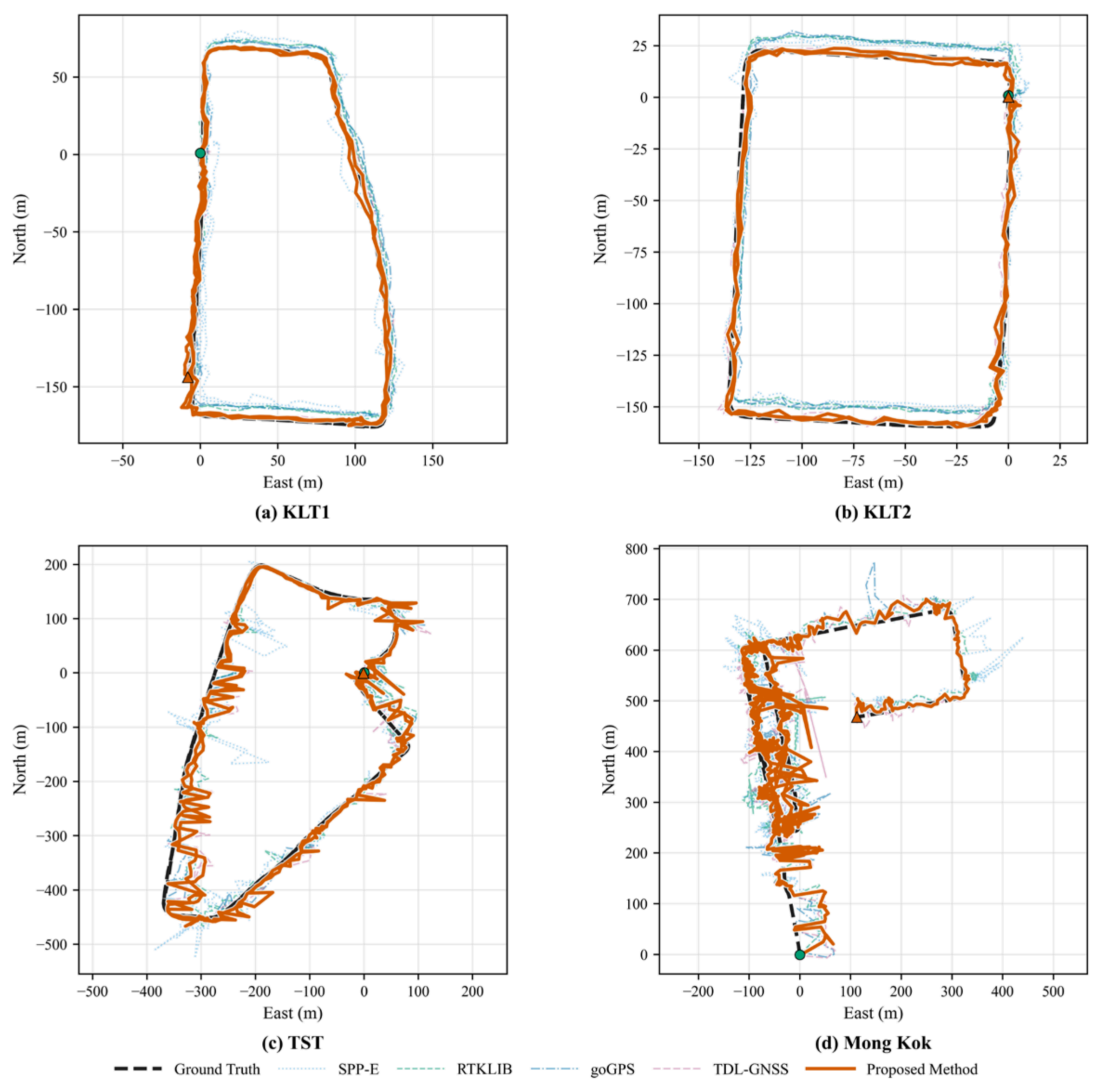
(**a**–**d**) Trajectory comparison across test sequences. The green circles and orange triangles indicate the starting and ending points of the ground-truth trajectories, respectively.

We present the estimated trajectories against the ground truth in Figure 9 to provide an intuitive illustration of the actual positioning enhancement. Four representative sequences are visualized, including KLT1, KLT2, TST and Mong Kok.

Figure 9a,b present the trajectory comparisons for the light urban canyon sequences KLT1 and KLT2. In both sequences, the proposed method follows the ground truth more closely than SPP-E, RTKLIB, and goGPS. On KLT1, the conventional methods show visible lateral offsets along the straight sections and around the upper and lower turns, whereas the proposed method remains closer to the reference route and produces a smoother trajectory. Compared with TDL-GNSS, the proposed method exhibits smaller overall offsets along the upper, right, and lower road sections, although minor local deviations remain near several turns. On KLT2, the advantages are more evident: the proposed method closely tracks the four sides of the rectangular route, with reduced lateral drift and fewer local fluctuations compared with the conventional methods. It also follows the ground truth more consistently than TDL-GNSS along the long straight sections and maintains better continuity around the corners. These observations demonstrate the effectiveness of the proposed method in improving trajectory accuracy, continuity, and stability under light urban canyon conditions.

Figure 9c presents the trajectory comparison for the TST sequence under medium urban canyon conditions. Although all methods exhibit increased deviations, the proposed method preserves the overall route shape and maintains continuous tracking throughout the sequence. It remains close to the ground truth along the upper curved section and the long lower-right section, while SPP-E and RTKLIB show more scattered deviations in several local areas. The proposed method maintains trajectory continuity comparable to goGPS and TDL-GNSS while remaining close to the ground truth along most sections of the route.

Figure 9d shows the results for the harsh urban canyon Mong Kok sequence. Severe signal degradation causes substantial fluctuations for all methods, particularly along the left vertical section. Nevertheless, the proposed method preserves the overall route structure and remains close to the ground truth along most sections. It exhibits fewer large, scattered deviations than the conventional methods and maintains a more bounded trajectory than TDL-GNSS in some severely obstructed sections.

Overall, the trajectory visualizations demonstrate that the proposed method can improve trajectory stability and continuity. In the light urban canyon scenarios, the proposed method follows the reference trajectory more consistently and exhibits fewer abrupt deviations, indicating improved stability in continuous positioning. In the more challenging TST and Mong Kok sequences, the method continues to preserve the overall route structure and constrain large trajectory deviations.

## 7. Conclusions

This article proposes a residual-guided hybrid stochastic modeling framework to mitigate GNSS positioning degradation in urban canyon environments. The proposed method follows a two-stage design: first, a pseudo-range correction estimation network predicts correction terms from multi-dimensional GNSS features to correct the pseudo-range observations; second, the estimated correction, together with elevation angle and C/N_0_, is introduced into a hybrid stochastic model parameter estimation network to determine adaptive satellite observation weights for weighted least-squares positioning. By jointly considering pseudo-range correction and adaptive stochastic modeling, the proposed framework integrates the interpretability of conventional stochastic models with the nonlinear representation capability of deep learning. Experiments on public urban GNSS datasets demonstrate that the proposed method achieves stable and competitive positioning performance across different urban scenarios. In the light urban canyon sequences KLT1 and KLT2, the proposed method achieves the lowest 2D mean errors of 2.66 m and 2.06 m, respectively, reducing the mean error by 61.1% and 71.0% compared with the best conventional baseline and by 32.5% compared with TDL-GNSS on KLT2. It also produces compact error distributions and follows the reference trajectories more consistently. In the TST sequence, the proposed method maintains competitive 2D and 3D positioning performance, despite being trained only in a light urban environment. In the harsh Mong Kok sequence, it achieves the lowest 2D 95th-percentile error and maintains favorable mean, median, and maximum 2D errors, demonstrating effective suppression of large positioning deviations. Its 3D maximum and 95th-percentile errors also remain comparable to those of the stronger baselines. These results verify the effectiveness of the proposed framework for urban GNSS positioning enhancement and demonstrate its cross-dataset applicability under different urban conditions. Nevertheless, the proposed method still has two main limitations. First, because the pseudo-range corrections are learned through the final positioning loss without satellite-level error labels, they cannot be interpreted as the true physical pseudo-range errors of individual satellites. Second, in complex urban environments with severe NLOS and multipath effects, positioning performance degrades and the model’s generalization capability requires further improvement. Future work will focus on observation-level error analysis and positioning accuracy enhancement under severe NLOS and multipath conditions.

## Figures and Tables

**Table 1:** Details of the experimental datasets.

Dataset	Date	Urban Canyon	Duration (s)	Usage
KLT1	11 September 2023	Light	421	Test
KLT2	11 September 2023	Light	294	Test
KLT3	11 September 2023	Light	666	Training
TST	17 May 2021	Medium	786	Test
Mong Kok	18 May 2021	Harsh	2312	Test

**Table 2:** Training details.

Component	Specification	
System	Windows 11	
CPU	AMD Ryzen 9 8940HX CPU	
GPU	NVIDIA GeForce RTX 5060 GPU	
Loss Function	L2 Norm of Position Error	
Epoch	100	
Optimizer	AdamW	
Optimizer Parameters	Learning Rate	5 × 10^−3^
	Weight Decay	1 × 10^−5^

**Table 3:** Comparison of 2D positioning errors (mean/max in meters).

	Dataset	KLT1	KLT2	TST	Mong Kok
Method					
SPP-E		9.51/16.73	7.58/14.14	20.78/145.54	23.68/142.76
RTKLIB		6.83/14.65	7.37/11.08	14.28/80.04	17.69/99.78
goGPS		7.36/11.26	7.11/11.00	9.23/64.24	12.48/119.24
TDL-GNSS		2.75/6.71	3.05/8.52	9.95/77.27	15.92/157.41
Proposed Method		2.66/7.23	2.06/6.04	11.14/78.93	13.93/100.12

**Table 4:** Comparison of 2D positioning errors at CDF levels (med/P95 in meters).

	Dataset	KLT1	KLT2	TST	Mong Kok
Method					
SPP-E		9.97/13.72	7.71/11.46	14.76/53.91	21.64/50.28
RTKLIB		6.16/13.46	7.26/9.45	7.24/53.19	14.86/40.91
goGPS		7.38/10.71	7.11/9.52	4.41/39.25	7.51/40.87
TDL-GNSS		2.51/5.54	2.88/5.80	5.52/48.47	11.12/48.32
Proposed Method		2.37/6.54	1.78/4.59	5.74/49.79	10.10/37.59

**Table 5:** Comparison of 3D positioning errors (mean/max in meters).

	Dataset	KLT1	KLT2	TST	Mong Kok
Method					
SPP-E		19.96/51.16	16.51/52.18	48.34/384.98	76.23/308.34
RTKLIB		17.52/39.74	13.86/37.78	33.39/166.34	60.78/333.56
goGPS		13.17/28.18	9.45/21.83	17.52/104.47	33.07/256.40
TDL-GNSS		4.95/19.19	5.09/11.03	17.49/104.58	33.59/332.54
Proposed Method		5.53/24.43	4.74/12.18	21.56/107.12	41.16/306.33

**Table 6:** Comparison of 3D positioning errors at CDF levels (med/P95 in meters).

	Dataset	KLT1	KLT2	TST	Mong Kok
Method					
SPP-E		16.86/38.51	14.55/31.35	40.02/121.81	71.41/148.16
RTKLIB		15.06/33.57	11.35/26.44	20.75/116.05	53.63/134.57
goGPS		12.18/23.65	8.78/15.44	9.21/62.94	24.26/87.92
TDL-GNSS		4.29/11.37	4.82/8.64	10.96/58.30	25.92/83.77
Proposed Method		4.03/14.86	4.28/9.77	13.12/70.67	32.74/89.79

**Table 7:** Ablation results of 2D and 3D positioning errors (mean/max in meters).

	Dataset	KLT1 (2D)	KLT2 (2D)	TST (2D)	Mong Kok (2D)	KLT1 (3D)	KLT2 (3D)	TST (3D)	Mong Kok (3D)
Method									
C/N_0_ Model		3.35/10.09	3.15/9.01	9.91/111.23	13.86/107.05	7.80/20.84	5.73/15.47	17.94/161.55	29.67/419.68
EL Model		3.03/8.97	2.40/9.89	12.78/72.15	16.93/100.12	6.12/35.76	7.40/27.78	23.66/133.45	51.19/306.33
Proposed Method Without Weight		5.37/15.99	3.75/9.58	17.54/103.72	22.05/107.60	12.45/73.99	9.48/27.48	34.13/292.11	59.40/282.98
Proposed Method Without Bias		7.70/12.65	7.15/12.46	11.18/110.79	14.37/104.13	14.20/30.17	9.50/21.79	24.04/127.84	44.35/327.54
Proposed Method		2.66/7.23	2.06/6.04	11.14/78.93	13.93/100.12	5.53/24.43	4.74/12.18	21.56/107.12	41.16/306.33

## Data Availability

The original data presented in this study are openly available in the RGHSM-GNSS repository at https://github.com/yzd-21/RGHSM-GNSS (accessed on 29 July 2026).
